# Uncertainty-aware deep learning for trustworthy prediction of long-term outcome after endovascular thrombectomy

**DOI:** 10.1038/s41598-024-55761-8

**Published:** 2024-03-06

**Authors:** Celia Martín Vicario, Dalia Rodríguez Salas, Andreas Maier, Stefan Hock, Joji Kuramatsu, Bernd Kallmuenzer, Florian Thamm, Oliver Taubmann, Hendrik Ditt, Stefan Schwab, Arnd Dörfler, Iris Muehlen

**Affiliations:** 1 Department of Neuroradiology, Friedrich-Alexander University of Erlangen-Nuremberg, University Hospital Erlangen, Erlangen, Germany; 2 Pattern Recognition Lab, Friedrich Alexander University, Erlangen, Germany; 3 Department of Neurology, Friedrich-Alexander University of Erlangen-Nuremberg, University Hospital Erlangen, Erlangen, Germany; 4 Siemens Healthineers, Forchheim, Germany

**Keywords:** Stroke, Predictive medicine, Machine learning, Outcomes research

## Abstract

Acute ischemic stroke (AIS) is a leading global cause of mortality and morbidity. Improving long-term outcome predictions after thrombectomy can enhance treatment quality by supporting clinical decision-making. With the advent of interpretable deep learning methods in recent years, it is now possible to develop trustworthy, high-performing prediction models. This study introduces an uncertainty-aware, graph deep learning model that predicts endovascular thrombectomy outcomes using clinical features and imaging biomarkers. The model targets long-term functional outcomes, defined by the three-month modified Rankin Score (mRS), and mortality rates. A sample of 220 AIS patients in the anterior circulation who underwent endovascular thrombectomy (EVT) was included, with 81 (37%) demonstrating good outcomes (mRS$\le$2). The performance of the different algorithms evaluated was comparable, with the maximum validation under the curve (AUC) reaching 0.87 using graph convolutional networks (GCN) for mRS prediction and 0.86 using fully connected networks (FCN) for mortality prediction. Moderate performance was obtained at admission (AUC of 0.76 using GCN), which improved to 0.84 post-thrombectomy and to 0.89 a day after stroke. Reliable uncertainty prediction of the model could be demonstrated.

## Introduction

Stroke remains one of the most formidable challenges in healthcare today, necessitating considerable resources for effective management. Approximately one in four individuals will develop this condition over their lifetime, establishing it as a leading global cause of mortality and morbidity^1^. Approximately 70% of strokes are acute ischemic strokes (AIS), resulting from the abrupt blockage of a cerebral artery. In recent years, endovascular thrombectomy (EVT)—mechanical clot removal through catheter angiography—has demonstrated significant disability reduction in selected AIS patients with large vessel occlusions. This holds when EVT is performed within 6 hours of symptom onset and up to 24 hours for patients selected using perfusion imaging^2–4^. Despite these advancements, around a third of surviving patients experience long-term disability^2^. Consequently, it is crucial to develop methods to mitigate this burden and improve the quality of life for affected individuals. The modified Rankin Score (mRS) is frequently employed to measure stroke functional outcomes^5^.

Predicting long-term outcomes following strokes is challenging yet integral to treatment selection, prognostic expectation management, and rehabilitation planning. It aids clinicians in identifying patients who would benefit from specific treatments and setting appropriate rehabilitation goals. Early approaches utilized traditional regression methods like logistic regression (LR) to predict ischemic stroke outcomes post-intravenous thrombolysis^6^. Since 2014, machine learning (ML) and deep learning (DL) have shown promising results^7–13^. Variables selected by experts, obtained using statistical tests, or identified automatically by ML or DL algorithms have been used to pinpoint independent predictors of good clinical outcomes^14,15^. However, most literature focuses on predicting functional outcomes post-intravenous thrombolysis, with few studies involving long-term outcome predictions post-EVT. Random forests, support vector machines, and neural networks have been employed to predict functional independence post-thrombectomy, defined as the 90-day mRS^16^. These studies found that functional independence could be predicted using baseline variables, but performance improved when treatment variables were included. Similar results have been achieved using extreme gradient boosting (XGBoost) and gradient boosting machines^17^. Conversely, while instance-based methods like k-Nearest-Neighbors have been employed in outcome prediction tasks^18,19^, they have not been applied to this specific task.

Existing methods do not consider the potential information embedded in the relationships between similar patients within their architectures. Such relationships could enhance predictive power by exploiting the similarities across patient groups. Data can be defined as a population graph where patients are represented as nodes and their relationships as edges, each carrying a distinct weight. Geometric deep learning (GDL) extends the capacity of DL to handle graph data. This approach, gaining significant attention in recent years, facilitates a superior representation of a wide range of data types^20^. When disease prediction is modeled using a graph, it becomes a node classification task. The utility of population graphs has been successfully illustrated in predicting Alzheimer’s disease, Autism disorder, and Multiple Sclerosis^21–24^.

Machine-assisted methods have shown promising results across various fields, including medicine. However, their lack of interpretability has restricted their integration into the medical domain. Additionally, the reluctance to entrust machines with life-critical decisions, potentially leading to severe consequences, poses another barrier^12^. Regardless, the potential applications of artificial intelligence (AI) in medicine are extensive and potentially transformative. This disparity between the potential benefits and the reluctance to fully implement AI methods has sparked interest in increasing the explainability and interpretability of models, thereby fostering trust in machine learning. The implementation of automated systems in this critical domain, particularly those without identifiable causal connections, necessitates the incorporation of uncertainty quantification (UQ) methods. If a model can assess the uncertainty associated with each specific case, it enables patients and clinicians to make safer, more effective decisions based on the model’s outputs. Predictions with high uncertainty may be disregarded or prioritized for detailed human assessment^25^. Therefore, accurate uncertainty estimates are crucial for enhancing the reliability of ML models^26,27^. Previous research has demonstrated that relying solely on the SoftMax function, commonly utilized in the final layer of neural networks, does not yield a direct estimation of model prediction uncertainty^28,29^. As an alternative, the Monte-Carlo dropout method has emerged as a cost-effective approach capable of furnishing reliable uncertainty estimates^24,30,31^. In this study, we investigate the effectiveness of employing Monte-Carlo (MC) dropout, drawing inspiration from Bayesian approximation techniques for uncertain predictions in convolutional neural networks (CNNs)^32^, to assess uncertainty within the context of population graph analysis. During training, dropout is randomly applied, and during inference, multiple stochastic forward passes are performed using random dropout on the population graph to estimate uncertainty from the prediction distribution. Given the prevalence of cerebrovascular diseases, the development of accurate yet trustworthy methods for predicting stroke outcomes post-EVT is critical. In this paper, we introduce, to the best of our knowledge, the first uncertainty-aware model for predicting outcomes post-mechanical thrombectomy for AIS. The main contributions of our work include:

- Geometric deep learning EVT outcome Prediction: We designed and implemented a GDL-based method for stroke outcome prediction post-EVT. We compared this new graph-based model with state-of-the-art algorithms and evaluated their performance at three distinct time points: upon admission, immediately post-treatment, and at a 24-hour follow-up.
- Uncertainty-aware EVT outcome prediction: We implemented and evaluated an uncertainty-aware method based on MC-Dropout to quantify uncertainty.
- Dataset statistical analysis: We conducted a statistical analysis of the dataset variables to better understand the data, identify independent predictors of good outcomes and mortality, and facilitate comparison of our results with other state-of-the-art works.

## Results

Out of 220 patients, 81 (36.8%) demonstrated good outcomes (mRS$\le$2), while 139 (63.2%) showed poor outcomes (mRS>2) after a three-month follow-up. Within the larger group, 42 patients (18.6%) were deceased within the three-month period (mRS$=$6).

### Univariate analysis

Table 1 enumerates the independent predictors of both favorable outcomes and mortality at the three-month follow-up. For the sake of interpretability, only variables with p-values less than 0.05, deemed significant in predicting either good outcomes or mortality, are displayed. Patients characterized by older age (on average 10 years), more severe strokes, and pre-existing disability from previous strokes are generally expected to have an unfavorable prognosis (see Table 1 in the Supplementary Material). An adverse prognosis is also noted in patients with atrial fibrillation and diabetes mellitus.

Several neuroimaging biomarkers derived from computer tomography (CT) perfusion independently predict favorable outcomes. For instance, the ASPECT Score, indicating the number of unaffected brain regions, median is 1 point higher in patients with favorable outcomes compared to those with adverse outcomes. The infarct core, denoting irreparably damaged brain tissue even after reperfusion, is on average 17 ml larger in patients with adverse outcomes. The core is determined by a cerebral blood flow measuring less than 30% of the volume of the contralateral hemisphere (CBF<30%). Improved collateral circulation is identified in patients with favorable outcomes, with the average TAN score 1 point higher in this group. The hypoperfusion intensity ratio (HIR, Hypoperfusion Index) and cerebral blood volume (CBV) Index, associated with infarct growth and collateral circulation, serve as additional indicators of a favorable prognosis. Conversely, an adverse outcome is linked to inferior reperfusion, indicated by a TICI score <2b, following EVT. Analyzing the follow-up CT conducted 24 hours post-stroke reveals that the ASPECT score averages 2 points higher and the stroke volume 30 ml smaller in patients exhibiting favorable outcomes compared to those with adverse outcomes.

**Table 1:** Univariate statistical analysis employed for the prediction of functional outcomes and mortality at 90 days; only variables with a p-value lower than 0.05, either in the prediction of functional outcome or mortality, are displayed.

	Good outcome (n$=$81)	Poor outcome (n$=$139)	p-value	Alive (n$=$178)	Dead (n$=$42)	p-value
Metadata						
Age	65.48 ± 12.38	74.73 ± 11.18	$<10^{-8}$	69.96 ± 12.49	77.14 ± 10.50	$<10^{-4}$
Clinical information						
NIHSS	12 [7–14]	17 [12–20]	$<10^{-6}$	13 [10–18]	19 [14–24]	$<10^{-6}$
Diabetes Mellitus	10 (12.3%)	37 (45.7%)	0.02	36 (20.2%)	23 (12.4%)	0.52
Atrial fibrillation	26 (32%)	71 (51.1%)	0.006	74 (41.6%)	23 (54.8%)	0.15
DOAC	3 (3.7%)	19 (8.6%)	0.03	14 (7.9%)	8 (19%)	0.13
Previous mRS	0 [0-0]	0 [0-1]	$<10^{-5}$	0 [0-1]	1 [0-2]	$<10^{-7}$
Image biomarkers						
E-ASPECTS	9 [8–10]	8 [6–10]	0.01	9 [7–10]	8 [6–10]	0.04
TAN score	2 [2–3]	2 [1–2]	$<10^{-6}$	2 [1–2]	1 [1–2]	0.01
COVES score	2 [1–3]	1 [0–2]	0.01	1 [0–2]	0 [0–2]	0.21
Clot burden score	7 [6–8]	7 [6–8]	0.02	7 [6–8]	6 [6–8]	0.06
CBF$<30\%$ (ml)	8.69 ± 17.78	25.04 ± 41.11	$<10^{-4}$	15.48 ± 30.69	34.02 ± 47.83	0.002
$T_\text {max}>6$s (ml)	125.2 ± 85.6	150.6 ± 94.1	0.05	133.7 ± 85.3	173.1 ± 110.5	0.01
Hypoperfusion Index	0.38 ± 0.23	0.47 ± 0.24	0.007	0.43 ± 0.23	0.5 ± 0.27	0.07
CBV Index	0.78 ± 0.13	0.72 ± 0.15	0.002	0.76 ± 0.13	0.68 ± 0.18	0.003
Treatment information						
TICI	3 [2b-3]	3 [2b-3]	0.01	3 [2b-3]	3 [2b-3]	0.05
SAE	9 (11.1%)	27 (19.42%)	0.16	20 (11.2%)	16 (38.1%)	$<10^{-5}$
Follow-up CT						
Infarct volume (ml)	27.01 ± 26.03	57.88 ± 49.74	$<10^{-7}$	39.94 ± 37.93	74.34 ± 60.55	$<10^{-6}$
ASPECT score	10 [9–10]	8 [5–10]	$<10^{-8}$	9 [7–10]	8 [4–9]	$<10^{-5}$
Time information						
Unknown onset	25 (30.9%)	65 (46.8%)	0.05	69 (38.8%)	19 (45.2%)	0.06
Time to intervention (min)	59.6 ± 22.5	67.2 ± 28.9	0.04	63.6 ± 25.6	48.23 ± 32.02	0.31

For numerical variables, the mean ± standard deviation is provided, categorical variables are expressed as median [IQR], while for binary variables, the frequency of occurrence within the given category is represented as a percentage (%). Key terms used include NIHSS (National Institutes of Health Stroke Scale), DOAC (direct oral anticoagulant), CBF<30% (cerebral blood flow less than 30% of the contralateral hemisphere), CBV (cerebral blood volume), TICI (thrombolysis in cerebral infarction), and SAE (severe adverse event).

Patients who succumbed within the three-month period were typically older, had experienced more severe strokes, and had suffered from prior strokes resulting in pre-existing disability. The median mRS of deceased patients was 1 with an IQR of [0–2], a relatively high value considering that only patients with a pre-stroke mRS of up to 2 were included. This finding suggests that patients with previous strokes and consequent disability are more likely to suffer a subsequent stroke with a fatal outcome. Moreover, the occurrence of a serious adverse event (SAE) during the intervention or the in-hospital stay, such as bleeding due to intravenous thrombolysis, serves as a strong mortality predictor. Mortality is also associated with inferior collateral circulation and a larger infarct core, as illustrated by an infarct volume averaging 34 ml larger, and an ASPECT score’s in median 1 point lower. Furthermore, an increased infarct volume and reduced ASPECT score on the follow-up CT are correlated with mortality.

### Prediction of morbidity and mortality

Table 2 delineates the primary outcomes for morbidity and mortality prediction at three distinct time points—admission, post-treatment, and follow-up—across all assessed algorithms. Additionally, a statistical analysis of the results is presented in Tables 2, and 3 in the Appendix. The results underscore that the performance of all classifiers progressively improves at subsequent time points, in parallel with the availability of additional information. At admission, for morbidity, a moderate prediction is achieved with an AUC reaching 0.79, using logistic regression. This result sees an enhancement with the inclusion of post-treatment variables, resulting in an increase of the AUC to 0.84 ($p_{value} = 0.04$). Upon the incorporation of follow-up information after 24 hours, the AUC further increases to 0.87 ($p_{value} = 0.02$). At follow-up, all algorithms exhibit commendable discriminatory power, with an AUC ranging from 0.81 to 0.87. The graph convolutional network and logistic regression provide the most optimal favorable-outcome prediction accuracy overall, with an AUC of 0.87 at the follow-up timepoint which, however, is not significantly superior to RF and FCN. Upon evaluating balanced accuracy (B. accuracy), LR outperforms other methods at follow-up, suggesting that the classifier performs well across both classes. However, this algorithm only proves to be significantly superior to FCN and RF. For mortality, the most accurate estimation is achieved using the fully connected network, with an AUC of 0.86. The performance at admission is moderate, with an AUC of 0.77 achieved using the LR algorithm . Post-treatment results indicate marginal improvement, with the highest AUC at 0.78 using the FCN ($p_{value} = 0.04$). Some algorithms show no performance enhancement at this time relative to admission, which suggests that either the reperfusion outcomes or the automatically selected variables hold limited predictive power for patient mortality within a three-month window.

**Table 2:** Morbidity and Mortality prediction results.

	Good outcome			Mortality		
	AUC	Accuracy	B. accuracy	AUC	Accuracy	B. accuracy
Admission						
Logistic regression	**0.79**	0.72	0.70	0.62	0.76	0.58
Random forest	0.75	0.70	0.67	**0.77**	0.80	0.67
Fully connected network	0.77	0.70	0.67	0.66	0.69	0.56
Extreme gradient boost	0.71	0.71	0.68	0.72	0.77	0.58
Graph convolutional network	0.76	0.75	0.74	0.74	0.73	0.61
Post-EVT						
Logistic regression	**0.84**	0.77	0.77	0.73	0.81	0.69
Random forest	0.82	0.76	0.75	0.75	0.80	0.66
Fully connected network	0.83	0.77	0.77	**0.78**	0.73	0.61
Extreme gradient boost	0.75	0.71	0.74	0.69	0.76	0.63
Graph convolutional network	0.82	0.76	0.74	0.73	0.74	0.62
Follow-up						
Logistic regression	**0.87**	0.80	0.80	0.79	0.84	0.76
Random forest	0.85	0.76	0.75	0.85	0.81	0.67
Fully connected network	0.85	0.77	0.76	**0.86**	0.81	0.73
Extreme gradient boost	0.81	0.76	0.75	0.81	0.80	0.62
Graph convolutional network	**0.87**	0.76	0.75	0.83	0.80	0.70

Results of 3-month morbidity and mortality prediction for the evaluated algorithms at three different time points: Admission, post-treatment, and follow-up. The best AUC result at each time point is highlighted for better interpretation.

### Uncertainty results

**Figure 1:**
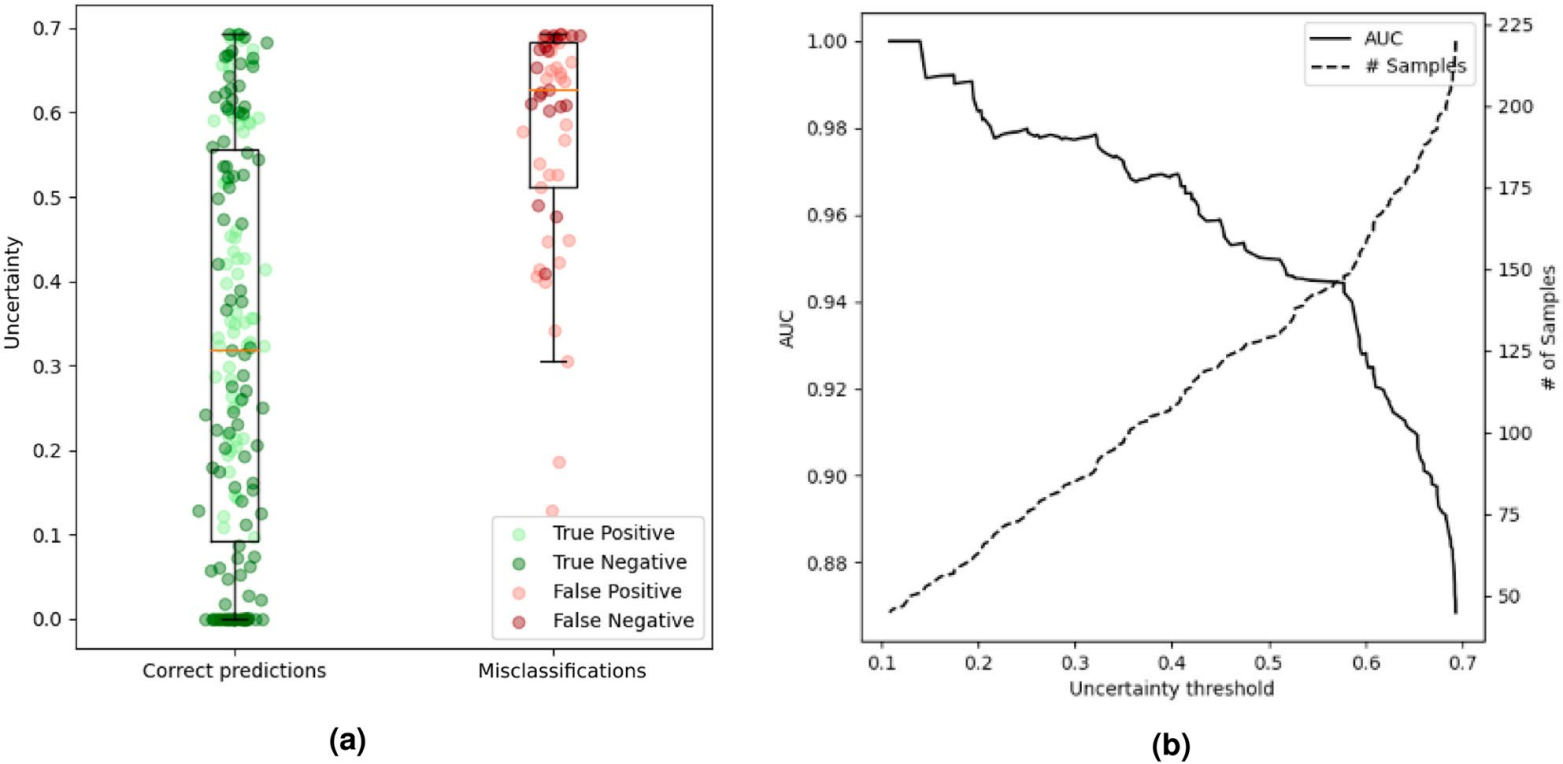
Morbidity prediction uncertainty quantification evaluation. (**a**) Boxplot representation of uncertainty value distribution in the correct prediction and misclassification groups. (**b**) Graph that shows the uncertainty threshold against the AUC and the number of patients accepted as certain.

Results for morbidity uncertainty quantification are presented in Fig. 1 and Table 3. Figure 1a illustrates the distribution of uncertainty predictions. The mean uncertainty is 0.32 for correctly classified patients, and 0.57 for misclassified patients. In our methodology, the uncertainty threshold (*th*) necessitates manual selection, contingent on the physician’s accuracy requirements for method adoption. As shown in Fig. 1b, the performance progressively escalates as patients with lower uncertainty values are included, and patients with higher uncertainty values are omitted in the model’s performance analysis. However, as the threshold decreases, the number of patients classified as uncertain increases. For patients whose uncertainty level exceeds the selected threshold, a priority should be placed on a detailed human assessment. The balance between accuracy and the number of patients with uncertain diagnoses influences the selection of an optimal threshold value. We assessed the performance and number of certain/uncertain patients using various thresholds. With a relatively high threshold value of $th$ $=$0.6, which designates 64 patients with the highest uncertainty values ($u_{pred}$=(0.6, 0.7]) as uncertain, the AUC achieves 0.92. A flawless classifier is obtained with a threshold of 0.1, which solely accepts the 43 patients with the lowest uncertainty values.

**Table 3:** Evaluation of results using different uncertainty thresholds.

Threshold ($u_{pred}<th$)	Good outcome				Mortality			
	# patients	AUC	Accuracy	B. accuracy	# patients	AUC	Accuracy	B. accuracy
0.1	43	1	1	1	38	1	1	1
0.2	62	0.98	0.97	0.9	72	0.96	0.96	0.87
0.3	84	0.98	0.98	0.95	103	0.92	0.93	0.83
0.4	107	0.97	0.95	0.93	127	0.89	0.90	0.78
0.5	128	0.95	0.90	0.88	149	0.86	0.83	0.78
0.6	156	0.92	0.87	0.85	174	0.86	0.89	0.79
0.7	220	0.87	0.76	0.75	220	0.83	0.80	0.70

The table shows for each threshold value, the number of predictions taken as certain, or in other words, the uncertainty value is lower than the given threshold. Additionally, the prediction results—both for morbidity and mortality—corresponding to each threshold value are displayed.

**Figure 2:**
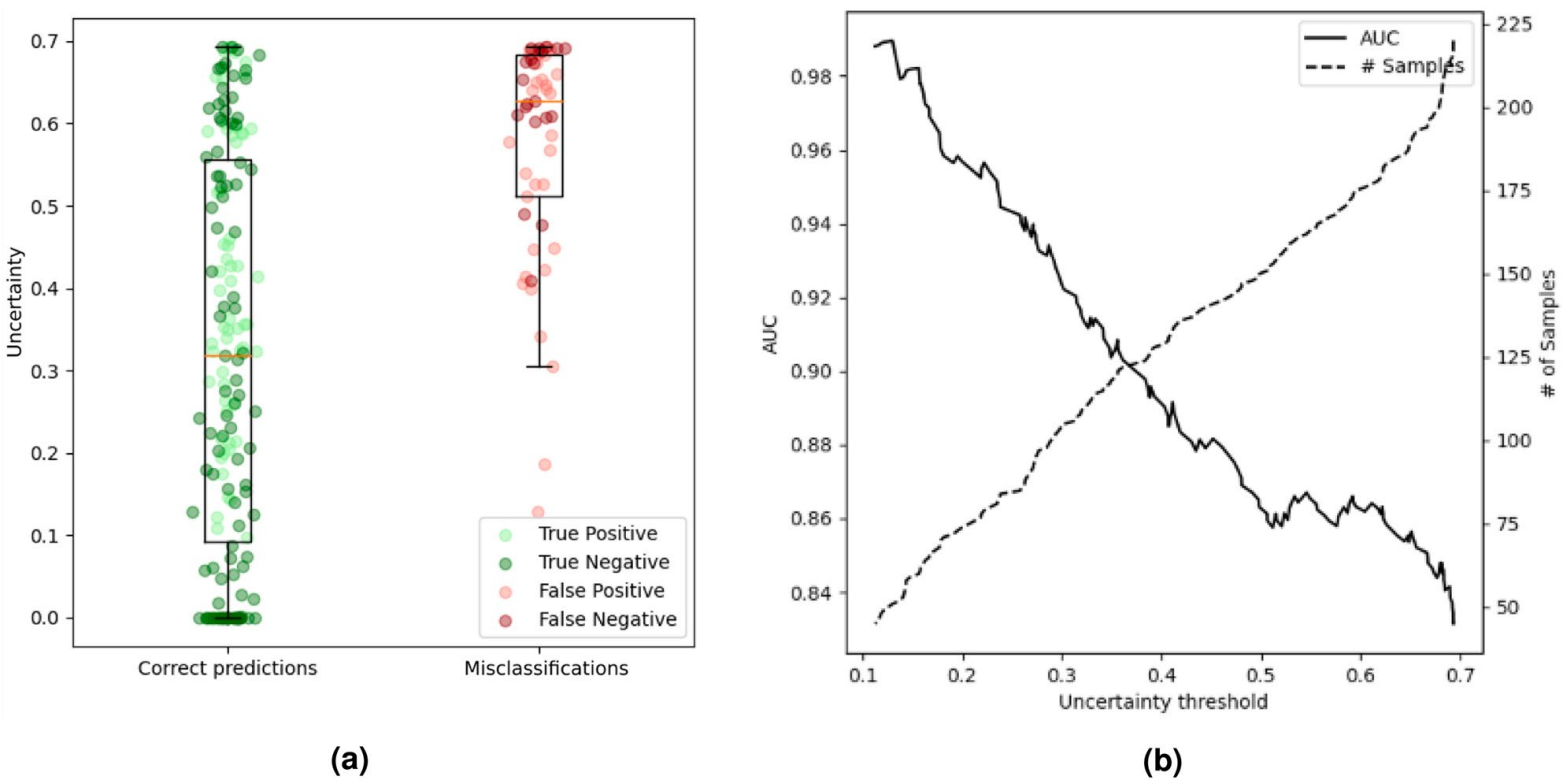
Mortality prediction uncertainty quantification evaluation. (**a**) Boxplot representation of uncertainty value distribution in the alive and dead within 3 months groups. (**b**) Graph that shows the uncertainty threshold against the AUC and the number of patients accepted as certain.

The results of mortality uncertainty quantification are presented in Fig. 2 and Table 3. The average uncertainty for correctly classified participants is 0.31, while the mean uncertainty for misclassified participants is 0.53. Upon examining the performance relative to the uncertainty threshold, we observe a progressive improvement as the threshold diminishes. In the context of mortality prediction, the area under the uncertainty/threshold curve is more compact, suggesting a lower uncertainty threshold is necessary to witness a substantial enhancement in the outcomes. By setting a threshold of 0.3, which designates the 117 patients with the highest uncertainty value as uncertain, the AUC reaches 0.92. A flawless classifier is similarly obtained with a threshold of 0.1, wherein certainty only applies to the 38 patients with the lowest uncertainty. The imbalance within the dataset affects these results, with the group of patients who survived and the group of patients with poor outcomes showing, on average, lower uncertainty.

## Discussion

This paper aims to introduce and evaluate an innovative approach for predicting long-term thrombectomy outcomes employing uncertainty-aware graph convolutional networks (GCN). We construct a population graph integrating demographic and clinical data, leveraging this structure to train a GCN for semi-supervised patient classification. Furthermore, we ascertain the uncertainty value associated with each prediction using the MC-Dropout method. The capability to predict long-term outcomes is crucial for refining EVT treatment strategies and patient selection, streamlining rehabilitation planning, and managing expectations for stroke patients and their families. Informed expectations enable physicians to devise personalized rehabilitation plans and provide better counsel to relatives. Deep learning methods could potentially enhance outcome prognoses after EVT for stroke patients. In our research, we conducted a comprehensive univariate analysis of 84 variables at three different time points, enabling the identification of several independent demographic, clinical, and imaging predictors for good functional outcomes and mortality. Upon admission, factors such as age, NIHSS, atrial fibrillation, and pre-stroke mRS significantly predicted outcomes, aligning with prior studies^14,15^. Furthermore, several parameters derived from CT angiography and CT perfusion were found to be independent predictors of the functional outcome. Good collateral status is associated with good outcomes, smaller infarct volumes, and lower incidences of hemorrhagic transformation following EVT^33–43^. The Hypoperfusion Intensity Ratio (HIR)-defined as the volume of tissue with Tmax>10s divided by the volume of tissue with Tmax>6s-has been associated with morphological collateral status and identified as a predictor of infarct progression and functional outcome, a finding corroborated by our results^44^. The cerebral blood volume index represents the mean CBV within the tissue volume exhibiting a Tmax>6s perfusion delay, divided by the mean CBV of healthy tissue. It has previously been delineated as a predictor of independent outcomes after thrombectomy, especially with low ASPECTS. This observation aligns with our study, wherein the CBV Index correlated with the modified Rankin Scale at 3 months and mortality^45^. Infarct core volumes, defined as relative cerebral blood flow (rCBF<30%), were significantly higher in patients with unfavorable outcomes and within the mortality group, confirming existing literature. However, the strength of this association may be modulated by clinical and therapeutic variables^46^.

A myriad of prediction models for functional long-term stroke outcomes have been established; conventional regression methods, such as logistic regression, were among the earliest approaches. However, since 2014, several machine learning techniques have demonstrated superior predictive power, owing to their ability to capture data non-linearities. The prevailing literature presents a mixture of findings, leaving it uncertain whether these techniques can outperform logistic regression^47^. Despite numerous studies indicating excellent performance of ML techniques, variances in the training setup, the patient sample size, and the variable origin complicate generalizations about ML algorithm efficacy. Such methodological discrepancies may contribute to inconsistencies in research findings. Using a cohort of 107 patients, Asadi et al. first proposed a dichotomized mRS ML-based prediction model for ischemic stroke, achieving an AUC of 0.6 and 0.7 using multilayer perceptron and random forest, respectively^7^. Heo et al., utilizing a prospective cohort of 2,604 acute ischemic stroke patients, found that ML-based models surpassed traditional approaches^9^. Ramos et al. used a dataset of 1,526 patients to predict poor outcomes post-EVT, ultimately concluding that, despite superior performance from other ML algorithms, logistic regression was the most effective strategy, considering its training time and interpretability^48^. Alaka et al. found comparable predictive accuracy between logistic regression and ML models in acute ischemic stroke patients 90 days post-EVT, based on both internal and external validation^11^.

In our study, in which we analyzed 220 patients using demographic, clinical, and imaging biomarker variables, we discovered that the highest-performing models, at follow-up, were the graph convolutional network and logistic regression, with an AUC of 0.87 for morbidity, and the fully connected network with an AUC of 0.86 for mortality prediction. However, all evaluated algorithms demonstrated significant discriminatory power. These findings emphasize the need for further research to assess the value of ML algorithms over traditional methodologies. As anticipated, classifier performance increased over time post-stroke onset as more parameters were added. This trend persisted across all studied algorithms. The predictive power at admission was moderate, with an AUC of 0.79 for morbidity and 0.77 for mortality. After treatment, the inclusion of recanalization results in substantially improved performance in morbidity prediction, with an AUC of 0.88. However, intervention results only marginally enhanced mortality prediction performance, likely due to the high rate of successful vessel recanalization and a very low rate of serious adverse events (SAEs) during the intervention. The after-treatment variables associated with morbidity were thrombolysis in cerebral infarction (TICI) score, number of maneuvers, and the device used, whereas SAE and TICI scores were associated with mortality. Although post-thrombectomy and follow-up results cannot be used prospectively to determine the best treatment, they are invaluable for informing physicians, patients, and relatives about potential rehabilitation options. Moreover, these results may be used to develop realistic rehabilitation plans that best meet the patient’s needs. All the algorithms performed exceptionally well at the latest time point when information from follow-up scans conducted 24 hours after stroke onset was incorporated. ASPECTS, unlike the precise infarct volume, can be readily evaluated on follow-up CT and has proven to be a valuable outcome predictor. This finding aligns with studies by Leker et al., who demonstrated that post-stroke ASPECTS predicts outcomes post-thrombectomy^49^. Our findings align with those from Monteiro et al. who predicted the functional outcome three months post-stroke in 425 patients treated with recombinant tissue plasminogen activator (rtPA)^8^. They achieved an AUC of 0.81 at admission, which rose to 0.92 after 24 hours. We hypothesize that the slightly enhanced performance in their study, compared to ours, may be attributed to a larger patient cohort and the consideration of additional features, such as laboratory findings. Additionally, rtPA has a lower recanalization rate compared to EVT. Therefore, a larger proportion of non-recanalizing patients in Monteiro et al. may have reduced the variation in outcomes compared to this study. The assertion that stroke outcome prediction accuracy improves over time is unsurprising and can be attributed to the fact that the extent of the stroke is less likely to change at later stages. Upon a patient’s admission with acute stroke, there exists salvageable brain tissue, which may or may not result in infarcted tissue, dependent on numerous factors, including the success of recanalization, the collateral circulation, or the patient’s age. At 24 hours post-stroke, the infarcted region generally stabilizes, and further growth is unlikely.

The objective of this study was to introduce and assess a novel method for predicting long-term thrombectomy outcomes using uncertainty-aware graph convolutional networks. By quantifying uncertainty via the MC-Dropout method-measuring discrepancies between predictions made by various models with randomly applied dropout during forward testing passes-we effectively contrasted mean uncertainty across correctly predicted and misclassified groups. In morbidity prediction, these mean uncertainties were 0.32 and 0.57, respectively, while in mortality prediction, they were 0.31 and 0.53. Our method allows clinicians to manually set the desired threshold, which could enhance trust and adoption of these advanced algorithms that are often perceived as ’black-box’ models. Notably, there is an inherent trade-off between model performance and the number of patients classified as uncertain. This balance can be achieved by carefully selecting a threshold value that best suits the clinical context. As the threshold value decreases, the AUC increases, albeit at the expense of fewer patients being classified with certainty. Intriguingly, with an uncertainty threshold of 0.1, our model achieved perfect classification for both morbidity and mortality predictions. Moreover, we observed that classes with larger subject numbers exhibited lower uncertainty, further validating the effectiveness of our uncertainty quantification method.

Clinical implementation of these predictive models could significantly advance the field of acute ischemic stroke prognosis and management. Given their potential to provide highly accurate predictions of patient outcomes following mechanical thrombectomy, these models could greatly assist clinicians in making informed decisions about patient treatment and care. However, this is not without limitations. It is important to acknowledge the intrinsic difficulty of incorporating such methods into clinical practice, considering the stringent regulatory requirements and the intrinsic challenges in defining clinical guidelines for treatment. Establishing a robust clinical framework, obtaining regulatory approvals, and developing clear guidelines are critical steps in realizing the practical application of these models in the complex landscape of stroke care. Moreover, by enabling the selection of a customized uncertainty threshold, these algorithms can be tailored to individual clinical settings and patient populations, enhancing their applicability in various medical contexts. Importantly, this uncertainty-aware GDL method does not merely provide black-box predictions, but it also quantifies the level of uncertainty associated with each prediction, thereby facilitating a more nuanced understanding of the prognosis. This could be instrumental in fostering more informed discussions among healthcare providers, patients, and their families about expected outcomes and potential rehabilitation plans.

This study is not without limitations. As a single-center, retrospective study with a relatively modest dataset, the requirements for large data volumes may impact the performance of our ML and DL models. The extensive variable dataset could potentially result in under- or overestimation of certain clinical variables. Further, the data cleaning and patient selection processes, specifically the exclusion of patients with a pre-stroke mRS score greater than 2 or unknown 3-month outcomes, could introduce bias. Notably, our study did not incorporate laboratory variables such as hyperglycemia or leukocytosis. Conversely, the main strength of our study lies in its novelty: we present, for the first time, an uncertainty-aware graph deep learning method for outcome prediction following mechanical thrombectomy in acute ischemic stroke patients. This approach enhances the interpretability of deep learning models and incorporates several neuroimaging variables extracted from multimodal CT images, such as ASPECT score, TAN score, HIR, CBV index, and mismatch calculations, which many previous studies overlooked^14–17^. Nevertheless, it its paramount to acknowledge that this approach entails the loss of information encoded in the original images. To address this limitation and further leverage the capabilities of deep learning in extracting task-optimized features, we plan to explore the direct use of CT images in future works. To achieve successful clinical integration, several challenges must be overcome. These include integrating the predictive models into existing health information systems, ensuring data privacy and security, and training healthcare professionals to effectively interpret and apply the predictions. Further validation of our results in larger, ideally multicenter datasets, would provide more precise outcome predictions and establish a stronger foundation for the generalization of these findings. Furthermore, concerted efforts should be made to address these challenges in order to facilitate the broader adoption and integration of these promising technologies within clinical practice.

## Conclusion

To our knowledge, this is the first instance of an uncertainty-aware graph deep learning methodology being employed to predict clinical outcomes following mechanical thrombectomy for acute ischemic stroke. Consequently, we have been able to forecast morbidity and mortality with performance comparable to other evaluated techniques. The efficacy of all algorithms was enhanced when variables from subsequent time points were incorporated, achieving a maximum validation under the curve AUC of 0.87 for dichotomized mRS prediction and 0.86 for mortality prediction. Our univariate analysis recognized several variables as independent predictors of favorable outcomes, the most significant being age, NIHSS, prior mRS, TAN score, and an array of neuroimaging factors derived from CT perfusion and follow-up CT. Employing the Monte Carlo dropout method, we successfully captured the uncertainty value in our trials. The uncertainty among the misclassified predictions, as compared to the correctly classified predictions, was 0.25 points greater in the morbidity prediction task and 0.22 points in the mortality prediction task.

## Materials and methods

### Patient selection

We conducted a retrospective review of data from 383 acute ischemic stroke (AIS) patients who underwent mechanical thrombectomy at a tertiary care neurovascular center between January 2015 and December 2019. The criteria for inclusion in this study were: (1) AIS in the anterior circulation, (2) a pre-stroke mRS score of $\le$ 2, and (3) a known clinical outcome at three months according to the mRS. Upon excluding patients with posterior circulation stroke (n$=$48), those with unknown outcomes at 90 days (n$=$62), and patients with a pre-stroke mRS score>2 (n$=$55), a total of 220 patients were included in the study. This study was performed in accordance with applicable guidelines and regulations. The use of this data was approved by the local ethics committee of the University Hospital of Erlangen. All participants or next of kin gave informed consent according to legal requirements.

### Imaging protocol

We used a 128-section scanner (Somatom Definition AS+; Siemens Healthineers, Forchheim, Germany). All patients underwent a multimodal imaging protocol that included non-enhanced CT (NECT), CT perfusion (CTP), and CT angiography (CTA). The decision to proceed with endovascular treatment was based on clinical data and imaging results, decided by an interdisciplinary consensus. Furthermore, a non-enhanced follow-up CT was performed 24 hours after the intervention. Baseline NECT was evaluated for early ischemic changes, as defined by the Alberta Stroke Program Early CT Score (ASPECTS, range 0-10). The site of occlusion was identified on the CTA. CTP was examined for the extent of the infarct core (defined as rCBF<30%), collateral status, the hypoperfusion index (HIR), and the CBV Index using automated CTP software (RAPID, iSchema View inc., Menlo Park, CA, USA). The extent and location of infarction were evaluated using semiautomated segmentation methods on follow-up CT scans performed 24 h after the intervention (syngo.via, Siemens Healthineers, Erlangen, Germany).

### Data cleaning and feature selection

The dataset, comprising 84 variables including clinical parameters, neuroimaging biomarkers, treatment, and time information, underwent data cleaning. Variables with over 10% missing values were removed. Missing values were inputed using the median value. Nominal categorical variables were encoded as one-hot vectors, while ordinal categorical variables were encoded as vectors with a length equal to the number of classes, preserving the ordering information. Numerical values were normalized to have a mean of zero and a standard deviation of one, with normalization performed separately on each data split. Post-cleaning, 81 variables remained, which we divided into three categories based on the acquisition time:

- Admission: metadata (age, sex), clinical information (e.g., atrial fibrillation, and pre-stroke mRS), clinical patient evaluation (e.g., NIHSS), neuroimaging biomarkers (e.g., ASPECT score, infarct volume, and collateral scores), and time from symptom onset to admission.
- After treatment: treatment information ( e.g., number of maneuvers, the device used), reperfusion results (e.g., TICI score, and frustrating recanalization), and time from the puncture to recanalization.
- Follow-up: results from follow-up CT performed after 24 hours (e.g. ASPECTS score, infarct volume).

Our primary outcome is the functional status at three months, measured by the mRS. This score is binarized into favorable outcomes (mRS$\le$2) and poor outcomes (mRS>2). Our secondary endpoint is the mortality at three months. This score is categorized into alive (mRS<6) and deceased (mRS$=$6) patients. The mRS utilized in our study for both endpoints was assigned by medical experts affiliated with the Neurology department at the University Hospital of Erlangen.

The minimum redundancy maximum relevance (MRMR) algorithm was used for automatic feature selection, aimed at minimizing the redundancy within the selected set and choosing variables most closely related to class tags^50,51^. It iteratively adds features to the selected set, maximizing a specific score while minimizing mutual information between variables already in the set. The f-score is used for relevance and the Pearson correlation for redundancy. Features were dynamically selected for each timepoint, leaving out patients from the test set. The list of the features selected is displayed in the Table 4 of the appendix. The number of selected features was set at ten, and a 5-fold hold-out cross-validation was used for training. The data was partitioned into 5 folds, with 4 used for training and one for testing. Results were calculated by averaging across the five folds. Furthermore, a validation set constituting 20% of the training set is used. Data split is done to maintain the overall class distribution across different folds.

### Metrics

In this work we used 3 metrics to evaluate the performance of the classification tasks:

- *Area Under the Curve (AUC)* is a widely adopted metric for binary classification tasks, offering a comprehensive measure of model’s ability to discriminate between positive and negative samples across different probability thresholds. It captures the trade-off between sensitivity and specificity, offering a robust assessment of model’s discrimination power. In our study, AUC is specially relevant for assessing the performance of our prediction model in scenarios since the class distribution is imbalanced.
- *Accuracy (Acc.)* evaluates the correctness of predictions by measuring the ratio of correctly classifies to the total number of samples. While accuracy is highly intuitive to interpret, its utility is limited in imbalanced datasets, which is the case in this study. Nevertheless, we include accuracy among our evaluation metrics to provide a straightforward measure of overall model performance.
- *Balanced Accuracy (B. Acc.)*, an extension of accuracy, designed to address imbalanced class distribution. It is computed by averaging the sensitivity and specificity, providing a more nuanced understanding of a model’s performance when dealing with imbalanced datasets.

### Machine learning

In our study, we assess the performance of four distinct algorithms: logistic regression, random forest, fully connected networks, and extreme gradient boost. These were chosen based on their prevalent usage and demonstrated effectiveness in stroke outcome prediction. We utilized the Python module Scikit-Learn for the implementation of all machine learning algorithms, except for the fully connected networks, which was implemented using the Pytorch library^52^. The optimal hyperparameter configuration was determined via grid search on the validation set, selecting a different set of hyperparameters for each instance of the model.

- *Logistic regression (LR)* is a straightforward yet powerful classification algorithm that employs the logit function to model the conditional probability of class output. Essentially, it determines the correlation between a collection of independent variables and a single dependent variable. This algorithm performs exceptionally well with linearly separable classes, while its implementation remains simple. It is important to note that in logistic regression to yield dependable results, careful selection and preprocessing of independent variables are required^53^. In our study we use the LR algorithm with an l2 norm penalty. The solvers, namely newton-cholesky and liblinear, along with regularization values (1000, 100, 10), were optimized via a grid search in the validation set.
- *Random forest (RF)* is an ensemble of decision tree classification models, often constructed using a technique known as “bagging”. Multiple decision trees are trained on different subsets of the data and then combined to produce an overarching prediction. This approach enhances predictive power and mitigates model overfitting^54^. making random forests frequently employed in real-world applications, such as medical diagnosis and financial forecasting. We used a grid search to optimize the hyperparameters across the following dimensions: number of trees (2, 5, 10, 100)), maximum number of features (log(number features), sqrt(number of features), fixed number between: 2,5,10,100), maximum depth of each tree (5, 10 or not set), bootstrap use, and optimization function (gini, information gain).
- *Fully connected networks (FCN)* represent a widely recognized type of artificial neural network, composed of a series of interconnected layers. These networks, inspired by the human nervous system, are built upon neurons that are organized into layers and interconnected to form larger networks^55^. The FCN can learn, adapt, and optimize its structure and behavior using the backpropagation algorithm. With their universal approximation capabilities and flexible structure, FCNs serve as efficient tools for data-driven, dynamic modeling, excelling in tasks such as pattern recognition and handling incomplete or noisy data. As such, FCNs have become popular across various fields, including healthcare. In this study, we used a network with 6 layers, with the size of the hidden layers being 128, 64, 32, 16, and 8 . The Rectified Linear Unit is used and the optimizer is selected between stochastic gradient descent and the Adam optimized. Additionally, the learning rate is optimized withing the values 0.1, 0.01, 0.001, and 0.0001.
- * Extreme gradient boosting (XGBoost)* is a supervised algorithm that combines an ensemble of weak learners to create a robust learner. This algorithm is founded on the sequential development of prediction models, with each model iterating on its predecessor to enhance performance^56^. The gradient descent algorithm is used to optimize the algorithm’s parameters. In our study, each learner of the XGBoost algorithm is a decision tree. The hyperparamters optimized for XGBoost are maximum depth (2,4, 6), subsampling ratio of training instances (0.6, 0.8 and 1.0), subsamppling ratio of columns (0.4, 0.6, 0.8 and 1.0), and the minimum loss reduction required to make a further partition on a leaf node of the tree (0.5, 1, 1.5, and 2.5).

### Statistical analysis and reproducibility

In our study, we employed descriptive statistics to compare demographic, clinical, and biomarker characteristics of patients across different outcome groups. Univariate analyses were performed between each categorical or continuous predictor variable and the two binary outcomes: functional outcome at 90 days and mortality. The ${\chi }^2$-test was used for categorical variables, while continuous variables were analyzed using a Student’s t-test. When multiple hypothesis tests were conducted simultaneously, the False Discovery Rate (FDR) correction method was applied, ensuring appropriate adjustment for multiple comparisons. For the descriptive analysis of the quantitative variables, results are presented as the absolute number for binary variables, as the median [IQR] for categorical values, and as the mean (± SD) for continuous variables. To compare the algorithms results we used a paired t-test with a significance level of $\alpha =0.05$. The primary metric for evaluating the performance of each model is the validation under the receiver operating characteristics (ROC) curve (AUC). To ensure an unbiased evaluation, we ensured that the longitudinal acquisitions of the same participants fell within the same fold.

### Population graph construction

Besides feature vector selection, another critical design decision for our model was the graph construction that models the interaction between patients. This construction is pivotal as it captures the underlying data structure and delineates the similarity between feature vectors. Accordingly, we represent the population as a sparse graph $\fancyscript {G} = \{ \fancyscript {V}, \fancyscript {E}, \fancyscript {W} \}$, where $\fancyscript {W}$ is the adjacency matrix that describes the graph’s connectivity, $\fancyscript {V}$ represents each of the graph nodes and $e \subset \fancyscript {E}$ is an edge of the graph. Our sparse population graph incorporates phenotypic information, aiming to offer additional insight into the similarity between two subjects. The adjacency matrix $\fancyscript {W}$ of the graph is defined as follows:

1 $$\begin{aligned} \fancyscript {W} (i,j) = \text {sim} (m_i, m_j) \delta _{i,j} \end{aligned}$$

where $\text {sim}$ is a measure of similarity between patients, defined as the correlation distance between the meta information vectors *m*, which are composed of age, sex, NIHSS, and previous mRS. These variables were selected based on their availability at admission and their significance as independent predictors of the primary outcomes. The delta function $\delta _{i,j}$ ascertains if two nodes of our graphs are connected and they are optimized for each graph. This function is defined for functional outcome prediction as

2 $$\begin{aligned} \delta _{i,j} \; (\text {mRS}) = \,{\left\{ \begin{array}{ll} 1 &{} \text {if} \quad | pre\_mRS_i - pre\_mRS_j |< 1 \quad \text {and} \quad | age_i - age_j | < 3 \\ 0 &{} \text {otherwise}. \end{array}\right. } \end{aligned}$$

And for mortality prediction as

3 $$\begin{aligned} \delta _{i,j} \; (\text {mort.}) = \,{\left\{ \begin{array}{ll} 1 &{} \text {if} \quad \text {sex}_i = \text {sex}_j \quad \text {and} \quad | age_i - age_j | < 4 \\ 0 &{} \text {otherwise}. \end{array}\right. } \end{aligned}$$

The conditions embedded in these functions are obtained by a grid search over a set of 4 variables: age, sex, NIHSS, and pre-stroke mRS.

### Geometric deep learning architecture and uncertainty estimation

We utilized the Chebyshev Convolutional (ChebConv) network, an efficient generalization of CNNs using tools from Graph Signal Processing^57^. This model extracts local and stationary features through graph convolutional layers while avoiding the use of Graph Fourier. The network comprises two hidden Chebconv layers of size 40 and 20 respectively, followed by rectified linear unit (ReLU) activation. The output layer is followed by a Softmax activation function, while cross-entropy is employed to calculate the loss over all the samples. The model was implemented using the Adam optimizer, with a learning rate of 0.001, and a weight decay of 0.001. The convolutions were performed using $3{rd}$ order Chebyshev polynomials. Unlike in other algorithms hyperameters were not tuned for each fold in order to reduce the risk of overfitting on the dataset. Nevertheless, the optimal model configuration, including the graph architecture and hyperparameters, was optimized over a grid search. The models were trained with early stopping. For the implementation, we employed PyTorch Geometric, a geometric deep learning extension library for PyTorch^52^ which achieves high performance by leveraging dedicated CUDA kernels.

Uncertainty is estimated using the MC-Dropout method. This approach quantifies the uncertainty as the discordance between the predictions generated by different models with randomly applied dropout during forward passes of testing. This disagreement is calculated as the standard deviation of the predictions done by the sampled neural networks. The uncertainty corresponding to a particular sample is defined as:

4 $$\begin{aligned} u_{pred}= \frac{1}{T} \sum {\mu _{i,c} \log {\mu _{i,c}}} \end{aligned}$$

where T is the number of forward passes during testing, while $\mu$ is the mean prediction vector for a given sample and class. This method is comparable to sampling from a variational family (Gaussian mixture), thereby approximating the true deep Gaussian process posterior^32^. Consequently, the distribution of predictions stemming from multiple forward passes in a dropout-enabled network approximates sampling of the Bayesian posterior of a deep Gaussian process, and the standard deviation of such a distribution is an estimate of predictive uncertainty. Throughout our uncertainty quantification experiments, an ensemble of models generated a distribution of predictions for each patient, using T$=$100 forward passes in a dropout-enabled network with a dropout rate of p$=$0.1. This method has demonstrated its effectiveness in estimating uncertainty values near the training distribution but may exhibit challenges when applied to out-of-distribution data, such as patients from external medical centers. This limitation is a recognized aspect of uncertainty quantification methods and caution should be exercised in scenarios where the data distribution deviates from the train set. In this work, the output of the uncertainty is not calibrated to reflect the likelihood of true prediction. The uncertainty metric ranges from 0 to 0.7, indicating how uncertain that model is about a certain prediction, with 0 being the highest certainty level and 0.7 the highest uncertainty.

### Supplementary Information

Supplementary Tables.

## Data Availability

The datasets generated and/or analysed during the current study are not publicly available due to data protection regulations but are available from the corresponding author on reasonable request. The source code used in this research is available in the GitLab repository at https://gitlab.cs.fau.de/gu47bole/ais.
