# Supplementary material for: Uncertainty-aware deep learning for trustworthy prediction of long-term outcome after endovascular thrombectomy
Source: Sci Rep. 2024 Mar 6;14:5544. doi: 10.1038/s41598-024-55761-8 (PMC10917742; doi:10.1038/s41598-024-55761-8)
Supplement: Supplementary file 1 — Supplementary Tables. [file 41598_2024_55761_MOESM1_ESM.pdf]

Supplementary information

Table 1. Univariate statistical analysis for prediction of functional outcome and mortality at 90 days. Variables are divided according to the acquisition origin. The variables are expressed as mean ± standard deviation for numerical values, for categorical variables, it is expressed as median [IQR], and the count for the given category (%) is shown for binary variables. NIHSS: National Institutes of Health Stroke Scale; OAC: Oral Anticoagulant; VKA: Vitamin K Anticoagulant; DOAC: Direct Oral Anticoagulant; ICV: Internal Cerebral Brain; COVE Score: Cortical Vein Opacification Score; CBF< 30%: Cerebral Blood Flow lower than 30% of the contralateral hemisphere; $T_{max} > 6$s: Time to Maximum bigger than 6 seconds; CBV Index: Cerebral Blood Volume Index; TICI Score: Thrombolysis in Cerebral Infarction Score; PTA: Tissue Plasminogen Activator; CBV: Cerebral blood volume; SAE: Severe adverse event.

	Good outcome (n=81)	Poor outcome (n = 139)	p-value	Alive (n=178)	Dead (n =42)	p-value
**Metadata**						
Age	65,48 ± 12.38	74.73 ± 11.18	$< 10^{-8}$	69.96 ± 12.49	77.14 ± 10.50	$< 10^{-4}$
Male	46 (56.8%)	61 (43.9%)	0.09	88 (49.4%)	19 (45.2%)	0.75
**Clinical information**						
NIHSS	12 [7-14]	17 [12-20]	$< 10^{-6}$	13 [10-18]	19 [14-24]	$< 10^{-6}$
Previous Stroke	9 (11.1%)	21 (15.1%)	0.52	22 (12.4%)	8 (19%)	0.38
Hypertension	61 (75.3%)	115 (82.7%)	0.25	145 (81.5%)	31 (12.4%)	0.37
Hyperlipidemia	28 (34.6%)	50 (36%)	0.95	68 (38.2%)	10 (23.8%)	0.11
Diabetes Mellitus	10 (12.3%)	37 (45.7%)	0.02	36 (20.2%)	23 (12.4%)	0.52
Atrial Fibrillation	26 (32.1%)	71 (51.1%)	0.006	74 (41.6%)	23 (54.8%)	0.15
Antiplatelet	26 (32.1%)	43 (30.9%)	0.91	52 (29.2%)	17 (40.1%)	0.3
Antiplatelet (mono)	23 (28.4%)	39 (28.1%)	0.98	46 (25.8%)	16 (38.1%)	0.23
Antiplatelet (dual)	3 (3.7%)	4 (2.9%)	1	5 (2.8%)	2 (4.8%)	1
OAC	3 (3.7%)	4 (2.9%)	1	5 (2.8%)	2 (4.8%)	1
VKA	3 (3.7%)	12 (1.3%)	0.26	12 (6.7%)	3 (7.1%)	1
DOAC	3 (3.7%)	19 (8.6%)	0.03	14 (7.9%)	8 (19%)	0.13
Previous mRS	0 [0-0]	0 [0-1]	$< 10^{-5}$	0 [0-1]	1 [0-2]	$< 10^{-7}$
**Image biomarkers**						
E-ASPECTS	9 [8-10]	8 [6-10]	0.01	9 [7-10]	8 [6-10]	0.04
Superficial Stroke	17 (21%)	37 (26.6%)	0.13	44 (24.7%)	10 (23.8%)	0.23
Volume e-ASPECTS	37.84 ± 27.36	45.34 ± 32.86	0.07	40.71 ± 29.63	49.81 ± 36.25	0.09
ICV Infarcted Side	196.1 ± 51.2	185.76 ± 45.70	0.13	90.02 ± 47.81	88.05 ± 49.30	0.82
ICV Contralateral side	238.4 ± 47.8	232.1 ± 50.9	0.37	233.5 ± 49.8	238.8 ± 49.9	0.54
ICV Index	0.82 ± 0.14	0.82 ± 0.2	0.77	0.83 ± 0.18	0.80 ± 0.17	0.47
TAN Score	2 [2-3]	2 [1-2]	$< 10^{-6}$	2 [1-2]	1 [1-2]	0.01
COVES Score	2 [1-3]	1 [0-2]	0.01	1 [0-2]	0 [0-2]	0.21
Clot Burden Score	7 [6-8]	7 [6-8]	0.02	7 [6-8]	6 [6-8]	0.06
Vessel Stenosis	14 (17.3%)	30 (21.6%)	0.55	35 (19.7%)	9 (21.4%)	0.97
Arterial Dissection	3 (3.7%)	3 (2.2%)	0.67	6 (3.4%)	0 (0%)	0.60
CBF< 30% (ml/cm^3)	8.69 ± 17.78	25.04 ± 41.11	$< 10^{-4}$	15.48 ± 30.69	34.02 ± 47.83	0.002
$T_{max} > 6$s	125.2 ± 85.6	150.6 ± 94.1	0.05	133.7 ± 85.3	173.1 ± 110.5	0.01
Mismatch Volume	116.5 ± 85.6	125.5 ± 88.4	0.46	118.2 ± 82.2	139.1 ± 105.9	0.16
Mismatch Ratio (inv.)	0.08 ± 0.15	0.14 ± 0.2	0.01	0.11 ± 0.17	0.18 ± 0.23	0.04
Hypoperfusion Index	0.38 ± 0.23	0.47 ± 0.24	0.007	0.43 ± 0.23	0.5 ± 0.27	0.07
CBV Index	0.78 ± 0.13	0.72 ± 0.15	0.002	0.76 ± 0.13	0.68 ± 0.18	0.003
**Treatment information**						
Stenting	6 (7.4%)	3 (2.2%)	0.08	7 (3.9%)	2 (21.4%)	0.68
PTA	5 (6.2%)	5 (6.2%)	0.58	6 (3.4%)	4 (21.4%)	0.1

Continued on next page

Table 1

	Food outcome (n=81)	Poor outcome (n = 139)	p-value	Alive (n=178)	Dead (n =42)	p-value
Number Maneuver	14 (17.2%)	1(21.6%)	0.12	35 (19.7%)	9 (21.4%)	0.09
Lysis i.a	2 (2.5%)	0 (0%)	0.13	2 (1.1%)	0 (21.4%)	1
Frustrated recan.	1 (1.2%)	7 (5.0%)	0.26	4 (2.3%)	4 (21.4%)	0.05
Vessel occl. after EVT	1 (1.2%)	9 (6.4%)	0.31	35 (19.7%)	9 (21.4%)	0.97
TICI	3 [2b-3]	3 [2b-3]	0.01	3 [2b-3]	3 [2b-3]	0.05
SAE	9 (11.1%)	27 (19.4%)	0.89	20 (11.2%)	16 (38.1%)	$< 10^{-5}$
Vessel occl. new area	29 (35.8%)	40 (28.8%)	0.31	55 (30.9%)	14 (33.3%)	0.93
New occl. treatment	22 (27.2%)	29 (20.9%)	0.32	39 (21.9%)	12 (28.6%)	0.64
Infarct new area	11 (13.6%)	21 (15.1%)	0.89	25 (14.1%)	7 (16.7%)	1
**Follow-up CT**						
Infarct Volume (ml/cm^3)	27.01 ± 26.03	57.88 ± 49.74	$< 10^{-7}$	39.94 ± 37.93	74.34 ± 60.55	$< 10^{-6}$
ASPECT Score	10 [9-10]	8 [5-10]	$< 10^{-8}$	9 [7-10]	8 [4-9]	$< 10^{-5}$
**Time information**						
Unknown Onset	25 (30.9%)	65 (46.8%)	0.05	69 (38.8%)	19 (45.2%)	0.06
Onset to Admission (min)	293.7 ± 300.5	323.4 ± 303.6	0.49	304.2 ± 296.9	346.8 ± 324.5	0.42
Time to Intervention (min)	59.6 ± 22.5	67.2 ± 28.9	0.04	63.6 ± 25.6	48.23 ± 32.02	0.31
Intervention time (min)	69.1 ± 46.3	74.93 ± 41.3	0.33	72.1 ± 44.3	75.5 ± 38.3	0.65
Time to Control (min)	947.4 ± 391.3	846.4 ± 400.6	0.13	905.1 ± 404.6	852.9 ± 371.2	0.31

Table 2. Results of Paired t-Test comparisons between algorithm performance at different timepoints. Paired t-Tests were conducted for two pairs of timepoints: comparing results using variables present at admission and after EVT, as well as vairables present at admission and at follow-up. The table presents results for the three evaluation metrics used in this study. Values lower than 0.05 are highlighted, indicating statistically significant differences.

	Good Outcome			Mortality		
	Admission to Post-EVT	Post-EVT to Follow-up	Admission to Follow-up	Admission to Post-EVT	Post-EVT to Follow-up	Admission to Follow-up
**AUC**						
LR	**0.04**	0.29	**0.02**	**0.04**	0.27	**0.03**
RF	**0.02**	0.55	0.25	0.97	**0.003**	**0.001**
FCN	0.3	0.33	0.06	**0.05**	0.17	**0.02**
XGBoost	0.19	0.11	**0.02**	0.32	**0.01**	0.10
GCN	0.15	0.16	**0.01**	**0.01**	0.62	**0.01**
**Accuracy**						
LR	0.09	0.29	0.08	0.12	0.22	0.09
RF	**0.008**	0.20	**0.012**	0.71	0.50	0.23
FCN	**0.04**	0.82	0.13	**0.04**	0.8	0.09
XGBoost	0.81	0.49	0.68	0.73	0.44	0.46
GCN	0.31	0.17	**0.03**	**0.01**	0.51	0.17
**Balanced Accuracy**						
LR	0.07	0.23	0.08	0.10	0.54	**0.04**
RF	**0.01**	0.36	**0.007**	0.73	0.63	0.41
FCN	0.06	0.98	0.11	**0.05**	0.55	0.01
XGBoost	0.68	0.43	0.70	0.21	0.55	0.25
GCN	0.37	0.20	**0.05**	0.07	0.88	0.10

Table 3. Results of Paired t-Test comparisons between algorithm performance at follow-up. Paired t-Tests were conducted between each pair of algorithms for each evaluation metrics used in this study.Values lower than 0.05 are highlighted, indicating statistically significant differences with a threshold of 0.05.

	Good Outcome					Mortality				
	LR	RF	FCN	XGB	GCN	LR	RF	FCN	XGB	GCN
**AUC**										
Logistic regression	-	0.30	0.17	**0.03**	0.22	-	0.09	**0.002**	0.26	0.23
Random Forest	-	-	0.53	0.18	0.88	-	-	0.53	0.57	0.41
Fully Connected Network	-	-	-	0.64	0.69	-	-	-	0.26	0.20
XGBoost	-	-	-	-	0.48	-	-	-	-	0.92
Graph Convolutional Network	-	-	-	-	-	-	-	-	-	-
**Accuracy**										
Logistic regression	-	0.87	**0.004**	0.07	0.18	-	0.28	0.26	0.10	**0.04**
Random Forest	-	-	0.14	0.19	0.38	-	-	0.81	0.63	0.43
Fully Connected Network	-	-	-	0.79	0.55	-	-	-	0.92	0.18
XGBoost	-	-	-	-	0.81	-	-	-	-	0.65
Graph Convolutional Network	-	-	-	-	-	-	-	-	-	-
**Balanced Accuracy**										
Logistic regression	-	0.74	**0.03**	0.07	0.17	-	0.36	0.39	0.09	0.07
Random Forest	-	-	0.10	0.19	0.33	-	-	0.41	0.91	0.68
Fully Connected Network	-	-	-	0.93	0.72	-	-	-	0.37	0.36
XGBoost	-	-	-	-	0.80	-	-	-	-	0.49
Graph Convolutional Network	-	-	-	-	-	-	-	-	-	-

Table 4. Variables (n=10) selected for the training and evaluation of the presented algorithms. These features were selected using the MRMR method

	Selected Variables
**Good outcome**	
Admission	E-ASPECTS, TAN Score, COVES Score, Clot Burden Score, Age, NIHSS, previous mRS, Diabetes Mellitus, Atrial Fibrillation, OAC
Post-EVT	TAN Score, COVES Score, CBF<30%, Stenting, Number Maneuver, TICI Score, Age, NIHSS, pre-mRS, OAC
Follow-up	Tan Score, Coves Score, Stenting, Vessel Occlusion after Recan., Infarct Volume (follow-up), E-ASPECTS Score follow up, Age, NIHSS, previous mRS, OAC
**Mortality**	
Adimission	E-ASPECTS, TAN Score, COVES Score, Clot Burden Score, Tmax >6s, Age, NIHSS, pre-mRS, Hypertension, OAC
Post-EVT	E-ASPECTS, TAN Score, COVES Score, Tmax>6s, Vessel Occlusion after EVT, Age, SAE, NIHSS, previous mRS, OAC
Follow-up	E-ASPECTS, Vessel Occlusion after EVT, TAN Score, COVES Score, PTA, SAE, E-Aspect follow-up, Age, NIHSS, previous mRS
